# Social Support Contributes to Outcomes following Distal Radius Fractures

**DOI:** 10.1155/2013/867250

**Published:** 2013-11-04

**Authors:** Caitlin J. Symonette, Joy MacDermid, Ruby Grewal

**Affiliations:** ^1^Division of Plastic Surgery, Schulich School of Medicine and Dentistry, University of Western Ontario, 1151 Richmond Street, London, ON, Canada N6A 3K7; ^2^Division of Orthopedic Surgery, Hand and Upper Limb Center, St. Joseph's Health Care, University of Western Ontario, 268 Grosvenor Street, London, ON, Canada N6A 4L6

## Abstract

*Background*. Distal radius fractures are the most common fracture of the upper extremity and cause variable disability. This study examined the role of social support in patient-reported pain and disability at one year following distal radius fracture. *Methods*. The Medical Outcomes Study Social Support Survey was administered to a prospective cohort of 291 subjects with distal radius fractures at their baseline visit. Pearson correlations and stepwise linear regression models (*F*-to-remove 0.10) were used to identify whether social support contributes to wrist fracture outcomes. The primary outcome of pain and disability at one year was measured using the Patient Rated Wrist Evaluation. *Results*. Most injuries were low energy (67.5%) and were treated nonoperatively (71.9%). Pearson correlation analysis revealed that higher reported social support correlated with improved Patient Rated Wrist Evaluation scores at 1 year, *r*(*n* = 181) = −0.22, *P* < 0.05. Of the subscales within the Social Support Survey, emotional/informational support explained a significant proportion of the variance in 1-year Patient Rated Wrist Evaluation scores, *R* ^2^ = 4.7%, *F* (1, 181) = 9.98, *P* < 0.05. *Conclusion*. Lower emotional/informational social support at the time of distal radius fracture contributes a small but significant percentage to patient-reported pain and disability outcomes.

## 1. Introduction

Distal radius fractures (DRF) are the most common type of acute wrist trauma [1–3]. Despite being common, predicting a patient's outcome following DRF remains challenging. Fracture management, whether non-operative or operative, is directed at restoring anatomical alignment and function [3, 4]. Interestingly, an acceptable anatomical result does not always correlate with improved patient outcomes in residual pain and disability, particularly in older patients [5–7]. The advent of a biopsychosocial model of health and disease [8] has led to the emergence of instruments, such as the Patient Rated Wrist Evaluation (PRWE), which measure DRF outcome in terms of pain and disability [9]. The understanding of the contributions of both biological and psychosocial factors has improved the surgeon's ability to predict fracture outcomes.

Baseline characteristics such as pre reduction radial shortening, education, injury compensation, and the presence of other medical comorbidities have been shown to predict patient-reported pain and disability 1-year following DRF [7, 10]. Unfortunately, these variables are not modifiable by hand surgery or rehabilitation interventions. Therefore, they can be used to develop a prognosis but not to affect the outcome potential. Elucidating variables that can be modified during the patient's management strategy have potential for optimizing outcome following DRF.

The importance of social support in improved health outcomes is well validated in patients with a variety of medical conditions including coronary atherosclerosis [11], cancer [12, 13], HIV [14], and stroke [15]. Higher levels of social support have also been linked to improved recovery of premorbid level of functioning in the hip fracture population [16–18]. The role of social support has not yet been studied in the distal radius fracture population. The current investigation has utilized a biopsychosocial model to assess whether improved levels of baseline social support are predictive of reduced pain and disability at 1 year after distal radius fracture. 

## 2. Methods

### 2.1. Overview of the Study

This study was a prospective cohort study evaluating the role of social support in patient-reported pain and disability at 1 year after DRF. 

### 2.2. Cohort Recruitment

Skeletally mature patients were recruited from the practices of nine fellowship-trained hand surgeons at a single tertiary referral center between January 2002 and January 2010. Inclusion criteria included any individual older than 18 years, with an extra- or intra-articular DRF. Exclusion criteria included patients who did not have a complete Medical Outcomes Study (MOS) Social Support Survey at baseline. All participants provided informed written consent and the study was approved by the local Health Sciences Research Ethics Board. 

### 2.3. Cohort Demographics and Injury Characteristics

At the baseline visit, patient demographic and injury characteristics were recorded. Demographic data included age and gender. Injury characteristics included whether the dominant or nondominant hand was injured, the overall energy of the injury (low (e.g., fall from standing height), intermediate (e.g., fall or trauma from a low velocity activity such as rollerblading), and high (e.g., trauma from high velocity such as a motor vehicle collision)), open versus closed fracture, the mechanism of injury (fall on ice or snow, fall on outstretched hand, or other), presence of injury compensation, and treatment of the fracture (nonoperative versus operative). In addition, DRF complications were documented at baseline, 3, 6, and 12 months.

### 2.4. Outcome Variable

The outcome variable was the PRWE [9] score 1 year following DRF. The PRWE is a 15-item questionnaire composed of three subscales: pain, specific activities, and usual activities. The total score of the PRWE, including all three sub-scales, can range from zero (no pain/disability) to 100 (maximal pain/disability). 

### 2.5. Independent Variables

#### 2.5.1. Questionnaires

The Medical Outcomes Study (MOS) Social Support Survey [19] was administered to a cohort of 291 subjects with DRF at their baseline visit. The MOS Social Support Survey is a multidimensional, self-administered tool encompassing questions addressing four functional support scales (emotional/informational, tangible, affectionate, and positive social interactions) and an additional item (someone to do things with to help you get your mind off things) [19]. Using the developers instructions, the scaled scores were transformed to a 0–100 scale using the formula [100 ∗ ((observed score − minimal possible score)/(maximum possible score − minimal possible score)] [19] with a higher score indicating greater social support. 

General health related quality of life (HRQoL) was assessed using the SF-12 Health Survey (version 2). The total score of the SF-12 v.2 can range from 0 (lowest possible health) to 100 (maximal possible health). The SF-12 v.2 physical (PCS-12) and mental (MCS-12) summary scores were used to represent these two components of health status in the current study; scores are based on population norms with higher scores representing a better health status [20].

#### 2.5.2. Radiographic Assessment

Pre- and posttreatment and final radiographic results were evaluated. The overall severity of the fracture was assessed according to the AO classification system [21]. An assessment was made about the final position of the healed fracture, indicating whether the alignment was acceptable or unacceptable. Unacceptable alignment was defined as any one of or combination of a radial inclination of <15 degrees, radial shortening of >3 mm, and/or >20 degrees a volar tilt or >10 degrees of dorsal tilt. 

### 2.6. Data Analysis

The demographics, injury characteristics, treatment, and complications were analyzed using descriptive statistics. A paired *t*-test was used to evaluate differences between the baseline and one-year SF-12 scores (physical and mental components). Pearson correlations and stepwise regression were used to identify predictors of patient rated pain and disability according to the PRWE [9] at 1 year after DRF. A stepwise multiple linear regression model was created to determine whether the total MOS social support score was predictive of the 1-year PRWE score and subsequently which of the MOS Support subscales predicted the 1-year PRWE score. The *F*-to-enter was 0.05 and the *F*-to-remove was 0.10. Age, gender, radiographic alignment, and complications were tested to rule out their effect as potential confounding variables. All statistical analyses were performed using SPSS Statistical Software v.17.0. 

## 3. Results

### 3.1. Participant Characteristics

Two hundred and ninety-one patients met the eligibility criteria to be included in this study. Participants ranged in age from 18 to 84 years, with a mean age of 56.1 ± 15.5 years. The majority of the study subjects (198/291, 68.0%) were female. The mean PCS-12 score was 37.2 ± 9.4 at baseline. The mean MCS-12 score was 50.2 ± 11.3 at baseline. Both mean PCS-12 (48.7 ± 9.5) and MCS-12 (54.1 ± 8.9) scores were improved (*P* < 0.05) at the one-year follow-up appointment. 

### 3.2. Baseline Injury Characteristics and Treatment

Approximately half the cohort (46.0%) injured their dominant hand. The mechanism of fracture varied between participants. Twenty-four percent reported a fall on ice or snow, 56.6% reported a fall from standing height, and the remaining 20.0% stated another mechanism of injury such as a motor vehicle collision, rollerblading accident, or a fall from a raised platform. Accordingly, the majority of fractures were low energy (68.0%) and only a minority were high energy (11.0%). For the study cohort, only a minority of patients were involved in a claim for injury for injury compensation (16/271, 5.9%).  Table 1 summarizes the baseline radiographic AO classification and treatment.

### 3.3. Radiographic Parameters and Complications

Half the cohort (52.9%) did not have an acceptable alignment on their final radiograph. Of the participants lacking an acceptable reduction, the majority (78.7%) had been managed nonoperatively. The overall complication rate over the one-year followup was 20.0%. Almost half of the complications (8.0%) were mild with symptoms only and no specific treatment required. Overall, 45 patients had a complication at some point during their followup, with 15 of these patients reporting complications at the one-year followup.

### 3.4. Regression Model

The first model (Model 1) included only the total MOS social support score in the stepwise regression. The total MOS social support score was found to be predictive of the 1-year PRWE score, explaining 4.3% of the variability. The next model (Model 2) included the total value of each of the MOS subscales including emotional/informational support, tangible support, affectionate support and positive social interaction, and the additional item (“someone to do things with to help you get your mind off things”). The Durbin Watson test statistic for the second regression model was 1.80, indicating that the residuals are not correlated. Only emotional/informational support was found to be predictive of the 1-year PRWE score, explaining 4.7% of the variability seen in the 1-year PRWE score (Tables 2 and 3). The total MOS social support score and emotional/informational support score had Pearson correlations of −0.22 and −0.26, respectively.

In order to determine if any other variables were confounding the relationship between social support and outcome, a subsequent regression analysis was performed to evaluate the following potential confounders: age, gender, malunion, and treatment complications. Age, gender, presence of complications, and radiographic alignment were not identified as confounders as they did not influence the role emotional/informational support played in predicting 1-year PRWE scores. This further supports the importance of emotional/informational support in all patients independent of the radiographic alignment and presence of malunion.

## 4. Discussion

This study identified that fracture outcomes fit within a biopsychosocial paradigm, since baseline emotional/informational support contributes to 4.7% of the variability of the pain and disability outcome scores of distal radius fractures at one year, regardless of age, gender, and final radiographic alignment. The remaining MOS Social Support subscales including tangible support, affectionate support, positive social interaction, and “someone to help you get your mind off things” were not predictive of 1-year PRWE scores. 

The benefit of improved social support, in particular emotional support, early in the course of treatment of an elderly cohort of individuals with hip fractures is well established. Shyu et al. [18] found in a Taiwanese study of 126 patients with hip fractures that higher levels of affectionate/emotional support at 1 month following hospital discharge resulted in better recovery of the patient's function in their activities of daily living (ADLs) and HRQoL at 6 months. Other studies on patients older than 60 years with hip fractures, show that increased social support, even through regular telephone contact with one's social network [22], resulted in increased walking ability [17, 22] and recovery of premorbid level of functioning [16] by 6 months to 1 year. In addition, Mortimore et al. [23] found that no social contact during the two weeks prior to sustaining a hip fracture was associated with 5x increased risk of death over the following two years, compared with patients who received daily contact during that time. 

The current investigation extends the relationship of emotional/informational support to improved patient-reported outcomes in the distal radius fracture population. One cannot necessarily assume that the relationship observed in hip fracture would also be present for distal radius fractures since hip fracture impairs mobility, whereas upper extremity fractures may not necessarily do so. However, since upper extremity function is important to tasks of daily life there is a theoretical rationale to assume the relationship could be similar. However, the studies examining social support in hip fractures use predominantly elderly cohorts who have received operative intervention. In the current study we included a broader cross-section of age, inclusion of both intra- and extra-articular DRF, and the primarily nonoperative treatment of patients in the current study. One might expect that younger patients would be less in need of social support. Despite the differences in composition of the study cohorts, the benefit of emotional/informational support is preserved. Further neither agen or gender was a significant moderator of this effect in multivariate analysis.

We do not know the mechanism by which social support impacts outcome in the DRF population. For example, social support might empower people to participate more actively in their recovery, or it might be that instrumental help allows people to focus on healthy habits like optimal nutrition and participation in regular therapy sessions. Another potential explanation is that positive perceptions of having social support are related to an optimistic personality trait that also influences self-reported assessment of function.

Previous studies of social support vary slightly depending on whether social support is quantified according to social network structures or assessed according to the patient's perception of the quality of social support received. A study by Queenan et al. [12] suggested that functional support (the communication of information, social companionship, and happiness with the quality of support) was more predictive of HRQoL than structural social support (the amount/frequency of support or connection with community resources) in a sample of 196 men with prostate cancer. The current investigation is aligned with these findings showing that emotional support (a proxy measurement of functional support) and not tangible support (a proxy measurement of structural support) is predictive of patient-report pain and disability following DRF. 

The role of social support has also been established in other pathologies. A prospective study including hospitalized patients with hip fractures (*n* = 84), stroke (*n* = 79), or post-myocardial infarct (*n* = 106) showed that medical factors and pre-morbid emotional support predicted less disability at 6 weeks after diagnosis [24]. Further, Angerer et al. [11] showed that outward expression of anger and low social support, independent of medication and other risk factors, resulted in the progression of coronary atherosclerosis according to standard angiography performed over a two-year follow-up period. Similar to the current study, Bajunirwe et al. [14] demonstrated that informational/affectionate support, and not tangible support, is correlated with improved physical and mental health scores in patients receiving antiretroviral therapy for HIV/AIDS in Uganda. The important role of social support across a variety of pathologies highlights the importance of utilizing a more holistic approach including psychosocial factors when examining patient outcomes.

Other research has also studied which factors are predictive of pain and disability according to the PRWE following DRF [7, 10]. When looking at the natural recovery of DRF, only a minority of patients continue to have pain and disability according to the PRWE at 1 year after fracture and only a minority of patients have minimal pain and disability at the 6-month mark [25]. In one study, injury compensation predicted 25% of the variability in the 6-month PRWE score following DRF [7]. In a subsequent investigation by Grewal et al. [10], injury compensation, education, and other medical comorbidities explained 16.7% of the variability of the 1-year PRWE score. The current investigation adds to this body of literature, by explaining an additional 4.7% of the variability in the 1-year PRWE score following DRF.

One of the limitations to the study was that only a minority (58/291, 19%) of participants reported having social support available “little” or “none of the time.” Thus, participant inclusion in the study was independent of the results of their baseline MOS social support scores. 

Future investigations are required to further inform our understanding of when and how social support affects outcomes and potentially whether interventions designed to maximize emotional/informational support at baseline can result in improved outcomes. The additional variance of 4.7% explained by emotional support may appear small statistically; however the finding that baseline emotional support has a measureable impact one year after DRF pain and disability indicates a long-term effect deserving of clinical attention. Qualitative research will be needed to inform how social support affects patients with DRF and what social supports may be of value if an intervention was designed. Future studies in the DRF population are needed to characterize the role of interventions, including both formal and informal methods, to maximize social support on reduced pain and disability as an outcome measure.

## 5. Conclusion

Using a biopsychosocial approach to DRF, the current investigation examined the relationship between social support and patient-reported pain and disability 1 year after fracture. Of all of the MOS Social Support subscales examined, emotional/informational support was found to explain 4.7% of the variance in patient-reported pain and disability at 1 year. At the initial visit following a distal radius fracture, surgeons should consider counseling their patient on the potential rehabilitative benefits of improved social support.

## Figures and Tables

**Table 1:** AO fracture classification and treatment.

AO type	
A (extraarticular)	34.4%
B (partial articular)	17.3%
C (complete articular)	48.2%
Treatment	
Cast (undisplaced)	34.4%
Cast, CR	37.5%
CR, percutaneous pinning, ±Exfix	8.2%
Open reduction, Exfix	1.0%
ORIF	18.9%

CR: closed reduction, Exfix: external fixation, ORIF: open reduction internal fixation.

**Table 2:** Social support and 1-year PRWE scores (model summary).

Model	*R*	*R* square	Adjusted *R* square	Std. error of the estimate	Change statistics				
					*R* square change	*F* change	df1	df2	Sig. *F* change
1	0.221^a^	0.049	0.043	19.034	0.049	9.271	1	181	0.003
2	0.229^b^	0.052	0.047	18.998	0.052	9.975	1	181	0.002

Dependent variable: 1-year PRWE.

^a^Predictors (constant), MOS SSS-overall score transformed to a 0 to 100 scale.

^b^Predictors (constant), MOS SSS-total subscale score for emotional/informational support.

MOS SSS: Medical Outcomes Study Social Support Survey.

**Table 3:** Social support and 1-year PRWE scores (coefficients).

Model	Unstandardized coefficients		Standardized coefficients beta	*t*	Sig.
	*B*	Std. error			
1					
Constant	36.132	6.601		5.474	0.000
MOS SSS-total score	−0.252	0.083	−0.221	−3.05	0.003
2					
Constant	35.159	6.074		5.788	0.000
MOS SSS-total subscale score for emotional/informational support	−0.564	0.178	−0.229	−3.16	0.002

Dependent variable: 1-year PRWE.

MOS SSS: Medical Outcomes Study Social Support Survey.

## References

[1] Larsen CF, Lauritsen J (1993). Epidemiology of acute wrist trauma. *International Journal of Epidemiology*.

[2] Roumen RMH, Hesp WLEM, Bruggink EDM (1991). Unstable Colles’ fractures in elderly patients. A randomised trial of external fixation for redisplacement. *Journal of Bone and Joint Surgery. British*.

[3] Wadsworth TG (1990). Colles’ fracture. Failure in management may cause permanent disability. *British Medical Journal*.

[4] McQueen M, Caspers J (1988). Colles fracture: does the anatomical result affect the final function?. *Journal of Bone and Joint Surgery. British*.

[5] Anzarut A, Johnson JA, Rowe BH, Lambert RGW, Blitz S, Majumdar SR (2004). Radiologic and patient-reported functional outcomes in an elderly cohort with conservatively treated distal radius fractures. *Journal of Hand Surgery*.

[6] Grewal R, MacDermid JC (2007). The risk of adverse outcomes in extra-articular distal radius fractures is increased with malalignment in patients of all ages but mitigated in older patients. *Journal of Hand Surgery*.

[7] MacDermid JC, Donner A, Richards RS, Roth JH (2002). Patient versus injury factors as predictors of pain and disability six months after a distal radius fracture. *Journal of Clinical Epidemiology*.

[8] Havelka M, Lučanin JD, Lučanin D (2009). Biopsychosocial model—the integrated approach to health and disease. *Collegium Antropologicum*.

[9] MacDermid JC, Turgeon T, Richards RS, Beadle M, Roth JH (1998). Patient rating of wrist pain and disability: a reliable and valid measurement tool. *Journal of Orthopaedic Trauma*.

[10] Grewal R, MacDermid JC, Pope J, Chesworth BM (2007). Baseline predictors of pain and disability one year following extra-articular distal radius fractures. *Hand*.

[11] Angerer P, Siebert U, Kothny W, Mühlbauer D, Mudra H, von Schacky C (2000). Impact of social support, cynical hostility and anger expression on progression of coronary atherosclerosis. *Journal of the American College of Cardiology*.

[12] Queenan JA, Feldman-Stewart D, Brundage M, Groome PA (2010). Social support and quality of life of prostate cancer patients after radiotherapy treatment. *European Journal of Cancer Care*.

[13] Corboy D, McLaren S, Mcdonald J (2011). Predictors of support service use by rural and regional men with cancer. *Australian Journal of Rural Health*.

[14] Bajunirwe F, Tisch DJ, King CH, Arts EJ, Debanne SM, Sethi AK (2009). Quality of life and social support among patients receiving antiretroviral therapy in Western Uganda. *AIDS Care*.

[15] Hilari K, Northcott S, Roy P (2010). Psychological distress after stroke and aphasia: the first six months. *Clinical Rehabilitation*.

[16] Cummings SR, Phillips SL, Wheat ME (1988). Recovery of function after hip fracture. The role of social supports. *Journal of the American Geriatrics Society*.

[17] Mutran EJ, Reitzes DC, Mossey J, Fernandez ME (1995). Social support, depression, and recovery of walking ability following hip fracture surgery. *Journals of Gerontology. Series B*.

[18] Shyu Y-IL, Tang W-R, Tsai W-C, Liang J, Chen M-C (2006). Emotional support levels can predict physical functioning and health related quality of life among elderly Taiwanese with hip fractures. *Osteoporosis International*.

[19] Sherbourne CD, Stewart AL (1991). The MOS social support survey. *Social Science and Medicine*.

[20] Ware JE, Kosinski M, Keller SD (1996). A 12-Item Short-Form Health Survey: construction of scales and preliminary tests of reliability and validity. *Medical Care*.

[21] Müller ME, Nazarian S, Koch P, Schatzker J (1990). *The Comprehensive Classification of Fractures of Long Bones*.

[22] Magaziner J, Simonsick EM, Kashner TM, Hebel JR, Kenzora JE (1990). Predictors of functional recovery one year following hospital discharge for hip fracture: a prospective study. *Journals of Gerontology*.

[23] Mortimore E, Haselow D, Dolan M (2008). Amount of social contact and hip fracture mortality. *Journal of the American Geriatrics Society*.

[24] Wilcox VL, Kasl SV, Berkman LF (1994). Social support and physical disability in older people after hospitalization: a prospective study. *Health Psychology*.

[25] MacDermid JC, Roth JH, Richards RS (2003). Pain and disability reported in the year following a distal radius fracture: a cohort study. *BMC Musculoskeletal Disorders*.

